# Deciphering the Role of Melatonin-Related Signatures in Tumor Immunity and the Prognosis of Clear Cell Renal Cell Carcinoma

**DOI:** 10.1155/2023/3077091

**Published:** 2023-02-14

**Authors:** Aimin Jiang, Chen Cai, Yiren Yang, Yankang Cui, Wenqiang Liu, Qingyang Pang, Zhenjie Wu, Bing Liu, Le Qu, Peng Luo, Anbang Wang, Linhui Wang

**Affiliations:** ^1^Department of Urology, Changhai Hospital, Naval Medical University (Second Military Medical University), Shanghai, China; ^2^Department of Special Clinic, Changhai Hospital, Naval Medical University (Second Military Medical University), Shanghai, China; ^3^Department of Urology, Affiliated Jinling Hospital, Medical School of Nanjing University, Nanjing, China; ^4^Department of Urology, The Third Affiliated Hospital, Naval Medical University (Second Military Medical University), Shanghai, China; ^5^Department of Oncology, Zhujiang Hospital, Southern Medical University, Guangzhou, China; ^6^Department of Urology, Changzheng Hospital, Naval Medical University (Second Military Medical University), Shanghai, China

## Abstract

**Methods:**

Adopting multiomics data from TCGA and other public datasets, we analysed the expression, mutation, and prognostic evaluation in multiple cancers. ccRCC patients were categorized into two subgroups by an unsupervised cluster algorithm: melatonin-pattern cancer subtype 1 (MPCS1) and subtype 2 (MPCS2). We then explored the immune microenvironment, immune therapy response, and tumor metabolic pathways between the two subtypes. The clinical characteristics, genomic mutation landscape, and molecular inhibitor response were further investigated. Finally, a melatonin regulator-related prognostic model was constructed to predict patient prognosis in ccRCC.

**Results:**

We found that melatonin regulators were dysregulated depending on distinct cancer types, which were associated with genomic variation. The two subtypes indicated different clinical characteristics and biological processes in ccRCC. MPCS2, an aggressive subtype, led an advanced clinical stage and poorer survival of ccRCC patients. The activated oncogenic signaling pathway and metabolic signatures were responsible for cancer progression in the MPCS2 subtype. The MPCS2 subgroup suggested a higher tumor mutational burden and immune dysfunction state, resulting in a lower response to immunotherapy. The copy number variations of MPCS2 were significantly more frequent than those of MPCS1. In addition, the two subgroups exhibited distinct drug responsiveness, with MPCS2 being less responsive to multiple drugs. Finally, we established a subtype biomarker-based prognostic risk model that exhibited satisfactory performance in ccRCC patients.

**Conclusion:**

Melatonin regulator-related features could remodel functional pathways and the tumor immune microenvironment through genomic mutations and pathway regulation. Melatonin regulator-associated molecular subtypes enhance the understanding of the molecular characteristics of renal cancer and can guide clinical treatment. Activating the melatonergic system axis may improve the effect of immunotherapy for ccRCC.

## 1. Introduction

In the latest Global Cancer Report, 431,000 renal cell carcinoma (RCC) patients were documented, with 180,000 renal cancer-related deaths worldwide [1]. It is estimated that 10 to 15 percent of all adult kidney cancer are RCC, and clear cell cancers represent the most common RCC histology. Early-stage ccRCC has a relatively good prognosis after surgery. However, prognoses for metastatic ccRCC are egregiously poor, and studies have shown that the 5-year overall survival rate is below 5% [2]. Approximately one-quarter of the patients have already developed metastases when the tumor is diagnosed, and 30% of patients with early-stage renal cancer will eventually recur and metastasize [3, 4]. Unlike other cancers, ccRCC shows insensitivity to chemotherapy and radiotherapy. Recently, novel targeted therapy, immunotherapy, and combined therapy have prolonged advanced ccRCC patient to a certain extent. However, only some patients respond well to the drugs, and most of them would develop resistance [5]. The classic AJCC staging system is commonly applied to make risk stratification of ccRCC patients, but the existence of tumor heterogeneity makes its accuracy need to be further improved [6, 7]. In recent years, molecular subtype-based staging systems have partially improved the accuracy and specificity of prognostic prediction. Unfortunately, inappropriate machine learning algorithms, insufficiently mined cohort information, inappropriate validation datasets, and most models fail to apply in clinical practice. Thus, it is still necessary to develop and validate appropriate molecular subtypes to decode the biological heterogeneity of ccRCC patients.

Recent studies have shown that circadian rhythm disturbances are strongly associated with increased susceptibility to several cancers [8, 9]. Previous research indicated that the melatonergic system is mainly involved in circadian rhythms, but recent studies also revealed its role in anti-inflammatory, antidepressant, antioxidant, and antitumor activities. The melatonergic system has been found to exert anticancer efficacy through activating apoptosis, regulating cell proliferation signaling, modulating survival signals, and inhibiting angiogenesis [10]. Furthermore, melatonin inhibits the transition from oxidative phosphorylation (OXPHOS) to aerobic glycolysis by regulating key enzymes and glucose transporters. He et al. found that melatonin can antagonize HIF-1*α*-controlled aerobic glycolysis by scavenging ROS [11]. A recent report indicated that melatonin increased OXPHOS and inhibited glycolysis in HNSCC, leading to accumulated ROS production, apoptosis, and mitophagy [12]. Proietti et al. revealed that melatonin downregulated the expression of MDM2 and enhanced p53 acetylation in MCF-7 cells [13]. Zhang et al. found that melatonin suppressed hypoxia-induced glioblastoma cells by inhibiting HIF-1*α*, thereby delaying tumor progression [14]. Lee et al. found that sorafenib combined with melatonin could target the expression of metastasis-associated protein 2 in human renal cancer cells and synergistically inhibit the invasive ability of tumor cells [15]. Prieto-Domínguez found that melatonin could enhance the sensitivity of hepatoma cells to sorafenib, which is closely related to the production of reactive oxygen species and mitophagy [16]. The use of melatonin-like drugs may increase the responsiveness of various cancers to immunotherapy or targeted therapy. However, the specific role of melatonin axis modulators in ccRCC remains largely unknown.

In this study, we examined melatonin-related signatures by multiple cancer analysis and facilitated the risk stratification of ccRCC patients, including clinicopathological features, functional enrichment analysis, immune infiltration profiles, genome mutation signature, and drug sensitivity analysis. Based on subtype characteristics, a robust risk model, named RCC-MP6, was also constructed and validated using external datasets. Our study may encourage combination therapy to further improve the therapeutic effect and prognosis of ccRCC patients.

## 2. Materials and Method

### 2.1. Cancer Dataset Collection and Preprocessing

The pipeline of our research is visualized in Figure S1. Multiomics data of human cancers including normalized expression profile, DNA methylation, and clinical information were retrieved from GDC-TCGA dataset [17]. An external RCC dataset, E-MTAB-1980 or JAPAN-KIRC, containing complete expression profile and prognostic data, was download from the ArrayExpress database.

### 2.2. Identification of Different Melatonergic System-Related Subgroups in ccRCC

We included all melatonin-related regulators from previous studies, which are summarized in Table S1. We applied the R package ConsensusClusterPlus and the expression profile of melatonin regulators to perform unsupervised remodeling analysis. The optimal number of clusters was closed through the principle passement score and cophenetic coefficient.

### 2.3. Enrichment Analysis of Differentially Expressed Genes

All differentially expressed genes (DEGs) were calculated based on the count matrix of MPCS1 and MPCS2 with the use of R package DEseq2. The detailed thresholds for up- or downregulated genes were as follows: adjusted *p* value < 0.01, abstract log fold change > 2. After identifying DEGs, the R package ClusterProfiler was employed to GO, KEGG, GSEA, and GEVA. The annotation files to decipher the biological influence of DEGs were downloaded from the MSigDB [18] and ConsensusPathDB [19] databases.

### 2.4. Deconvolution of Immune Components and ICI Treatment Difference

We adopted seven immune-related deconvolution algorithms to quantify cellular components or immune cell enrichment scores. Furthermore, we utilized single sample-based GSVA to verify such differences [20–23]. The immune and stromal scores of each ccRCC patient were evaluated with the use of the R package ESTIMATE. Immunotherapy response sensitivity and immune exhaustion scores were calculated by TIDE algorithm [24].

### 2.5. Mutation Spectrum Characteristics among Subpopulations

We utilized the R package Maftools to compare subgroup's mutation landscape [25]. The detailed differences in exclusive or coexisting mutation patterns, onco-pathways, and druggable targets of mutation data were also analyzed [26]. For the analysis of gain and loss event at genome, the CNV were analyzed through the GISTIC 2.0 [27] algorithm which was calculated by Euclidean distance [28].

### 2.6. Drug Susceptibility Prediction

Three public pharmacogenomic databases, including GDSC, CellMiner [29], and CCLE [30], were applied to compare the half-maximal inhibitory concentration (IC50) and screen potential therapeutic agents for MPCS1 and MPCS2. For therapy response differences, we utilized the R package pRRophetic to estimate and cross-verify the results.

### 2.7. Construction and Verification of a Melatonin-Related Risk Model

We first calculated each subtype's biomarkers based on principle analysis and filtered prognostic signatures via univariate Cox regression analysis in TCGA-KIRC. The RSFVH was unutilized to decrease the model's redundancy. Last, a novel prognostic risk model maintaining a minimum number of signatures and pivotal features was screened. JAPAN-KIRC cohort was employed to validate the reproductivity of this model constructed in TCGA-KIRC.

### 2.8. Validating Different Expression Levels of ACHE

RT–qPCR was utilized to identify ACHE expression in tumor and adjacent renal tissues (including 22 ccRCC tissues from Changhai Hospital). The primer sequences for ACHE were forward primer: GGGTGGTAGACGCTACAACC and reverse primer: GTGCCCTCAAAACCTGGGTAT. For the primer sequences of GAPDH, refer to our previous study [31].

### 2.9. Statistical Analysis

All omics dataset processing and statistical analysis were finished by R software (version 4.1.3). For quantitative variables, Kruskal-Wallis and Wilcoxon tests were applied; for qualitative characteristics, the chi-square test was employed to compare differences. Correlations among variables were based on the R package corrplot. The R packages survival and pROC were used to plot Kaplan-Meier and time ROC curves. All two-sided *p* values (<0.05) were considered statistically significant between subtypes. The details for bioinformatics analysis can be found in previous studies [32–34].

## 3. Results

### 3.1. Dysregulation and Prognostic Significance of Melatonin-Related Genes in Cancers

**Figure 1:**
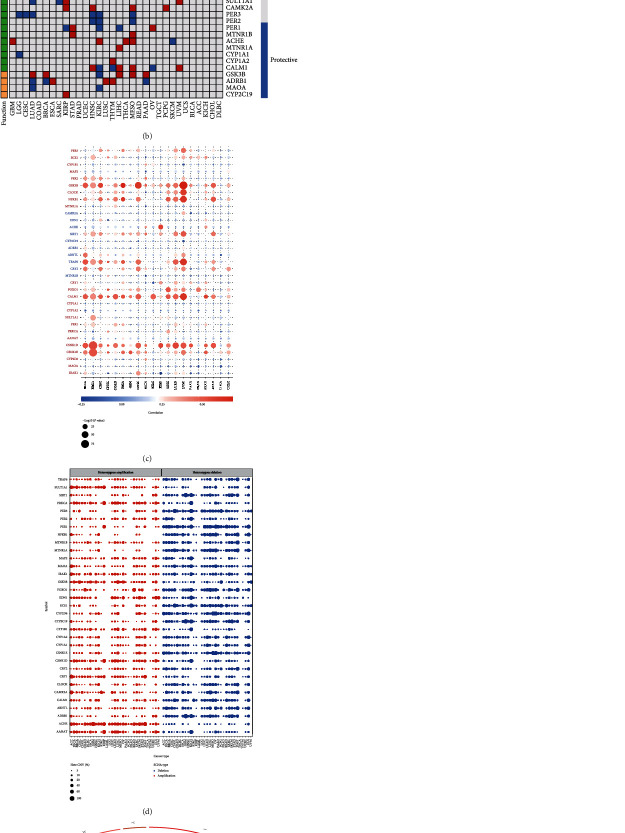
Dysregulation and mutation profile of melatonin-associated regulators across cancers. (a) The relative gene expression of melatonin-associated regulators. (b) The impact of melatonin-associated regulators on patient survival. (c) The correlation of CNV and the gene expression level of melatonin-associated regulators. (d) Heterozygous amplification or deletion of melatonin-associated regulators. (e) The genome locations of melatonin-associated regulators.

The workflow is depicted in Figure S1. To comprehensively investigate the role of the melatonergic system axis across cancers, we analyzed the detailed expression patterns of melatonin-related genes across cancers. Most melatonergic system axis modulators were upregulated in various cancers (Figure 1(a)). Survival analysis suggested that nearly all melatonin modulators were correlated with different patient outcomes depending on distinct cancer types (Figure 1(b)), indicating that dysregulated melatonin regulators played a crucial role in tumor progression. Interestingly, many melatonin-related regulators analyzed were protective factors for ccRCC, such as the CLOCK, FOXO1, and PER families.

To further explore the rationale of melatonin perturbation, we investigated the CNV and SNV of melatonin-related modulators across cancers. The expression of some melatonin genes, including GSK3B, NFKB1, CALM1, CSNK1D/E, and CLOCK, was positively related with CNV (Figure 1(c)). Heterozygous amplifications frequently appeared in SULT1A1, PRKCA, GSK3B, CRY1, ACHE, and AANAT, while heterozygous deletions often appeared in PER1, MTNR1A, FOXO1, ECE1, CYP2D6, and ADRB1 (Figure 1(d)). In ccRCC, we found compelling CNV gain for CAMK2A, while PER2, PER3, ECE1, MAP2, and MTNR1A were dominantly CNV loss (Figure S2A).  Figure 1(e) shows the genome location of melatonergic system axis regulators on chromosomes. We then inspected the SNV frequency of melatonin modulator genes, and 974 (61.1%) samples were altered in all tested samples (Figure S2B). Several genes were frequently mutated including MAP2, PER2, PER3, CYP2C19, PER1, and ECE1. Cancers with a higher SNV burden comprised UCEC, SKCM, COAS, STAD, LUSC, LUAD, BLCA, and CESC (Figure S2C). The dysregulation of melatonergic system axis modulators induced by genome alterations led to tumor progression in multiple cancers.

### 3.2. Two Distinctive ccRCC Subtypes Based on Melatonin Regulator Expression Pattern

**Figure 2:**
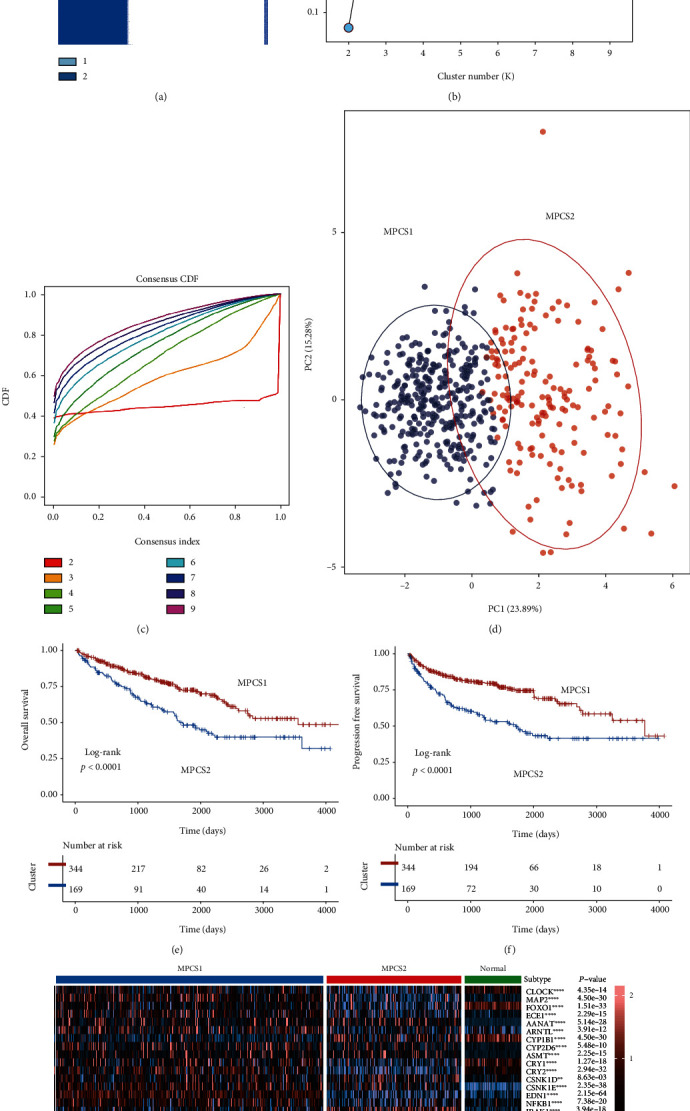
Identification of two clusters based on melatonin-related signatures. (a) Consensus cluster matrix of melatonin-related regulators in TCGA-KIRC. (b) The proportion of ambiguous clustering scores illustrating the optimal number of clusters. (c) CDF curves of the hierarchical model. (d) Two-dimensional principal component plot based on melatonin regulators. The blue dots represent MPCS1, and the red dots represent MPCS2. (e, f) Survival analysis of OS and PFS. (g) Heatmap of the expression of melatonin regulators in MPCS1, MPCS2, and normal tissues.

Melatonin regulators are not only correlated with circadian rhythms but also strongly associated with tumor progression. To investigate the specific role of melatonin modulators in ccRCC, we adopted the classic unsupervised clustering algorithm to perform remodeling analysis of TCGA-ccRCC. We chose *k* = 2 as the optimal cluster number through the R package and confirmed it using the PAC algorithm. ccRCC samples were specifically classified into two distinct subgroups, termed as MPCS1 and MPCS2 (Figure 2). The clinicopathological analysis found that MPCS2 patients showed a higher TNM stage and shorter survival than MPCS1 patients (Table S2, Figures 2(d) and 2(e)). Aside from that, we examined the expression of genes that modulate melatonin in MPCS1, MPCS2, and normal kidney tissues. MPCS2 was considered to be a suppressive phenotype of the melatonergic system axis, and its expression level of melatonin-related genes was significantly lower than that of MPCS1 and normal tissues. Of interest, CLOCK, FOXO1, CRY1/2, SIRT1, PER1/2/3, and CALM1 were significantly downregulated in MPCS2, while IRAK1, SULT1A1, and ACHE were upregulated in this subtype. Traditionally, the melatonin signaling axis has been primarily thought of as a tumor suppressor. The desert of the melatonin regulator axis in MPCS2 may partly induce an aggressive molecular subtype.

### 3.3. Functional Enrichment Analysis of ccRCC Subtypes

**Figure 3:**
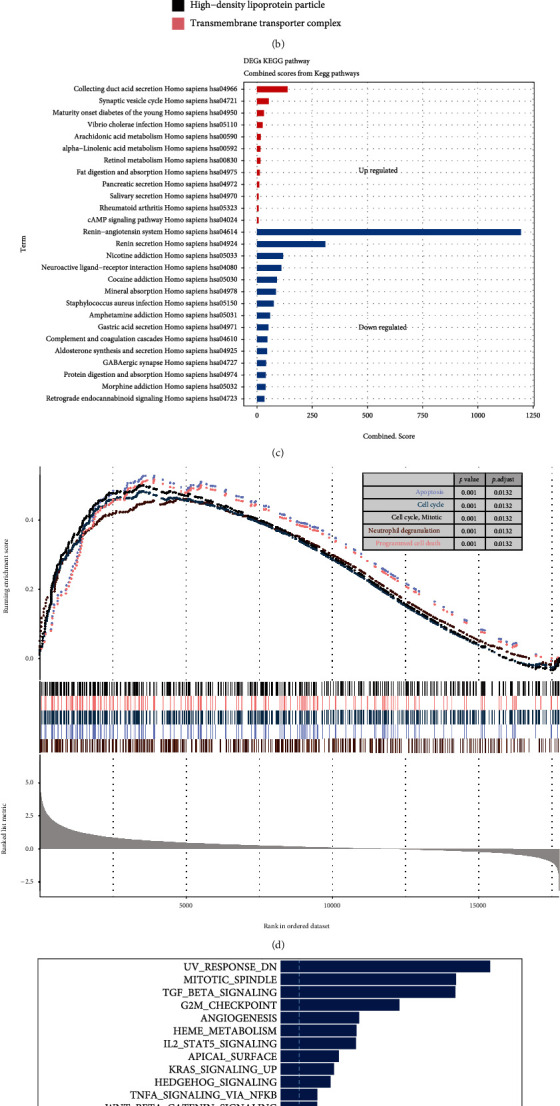
Various functional enrichment analyses of ccRCC subtypes. (a) Volcano plot indicating DEGs. (b) GO enrichment analysis of cellular component. (c) KEGG, (d) GSEA, and (e) GSVA between subtypes. (f) Regulation scores of different transcription factors. Yellow represents activated expression of transcription factors. Blue represents repressed expression of transcription factors.

Considering the subtype heterogeneity, we analyzed differences in expression profiles between the two subtypes (Figure 3(a)). We also performed functional enrichment analysis and identified a transcriptional regulatory network between them. GO enrichment analysis indicated that DEGs were primarily enriched in classic RCC-related onco-pathway, including collagen-containing extracellular matrix, high-density lipoprotein particle and ion channel complex in cellular component, acute-phase response and acute inflammatory response in biological process, and channel activity and passive transmembrane transporter activity in molecular function (Figure 3(b) and Figure S3A). The KEGG analysis indicated that collecting duct acid secretion and the synaptic vesicle cycle were significantly energized in MPCS2, while the renin−angiotensin system, renin secretion, and neuroactive ligand−receptor interaction were repressed in MPCS2 (Figure 3(c)). GSEA suggested that DEGs were predominantly involved in apoptosis, cell cycle, neutrophil degranulation, and programmed cell death (Figure 3(d)). Compared with MPCS1, GSVA showed that MPCS2 was activated in the UV response, mitotic spindle, TGF-BETA signaling, G2/M checkpoint, and angiogenesis and was repressed in peroxisome, coagulation, cholesterol homeostasis, glycolysis, and KRAS signaling (Figure 3(e)).

In addition, GSVA algorithms were also performed to explore other signatures, which indicated a repressive methylation phenotype and activated exosome secretion and metabolic activity in MPCS2 (Figure S3B). Of interest, MPCS2 was activated in retinoic acid metabolism, amino sugar metabolism, oxidative phosphorylation, glutathione metabolism, etc. The activated signaling indicated that melatonin-regulated axis participated in tumor progression from genomic, epigenetic, to metabolic (Figure S3B). Besides, the R package RTN was utilized to compare transcription factor activity between the two subgroups. We observed that ZEB2 and EPAS1 showed lower activity in MPCS2, suggesting suppression of EMT and sensitivity to hypoxia in the MPCS2 subtype (Figure 3(f)).

### 3.4. Immune Infiltration Profiles between the Two Subtypes

**Figure 4:**
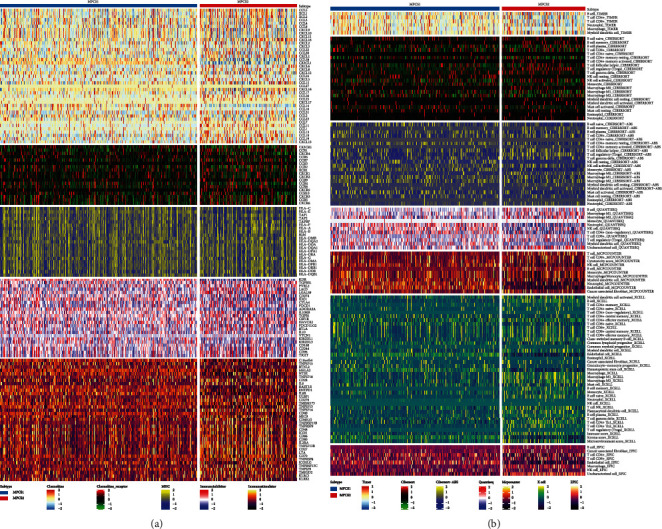
Immune profiling between subtypes. (a, b) Heatmap indicating the different immune signatures and immune component enrichment between subtypes.

**Figure 5:**
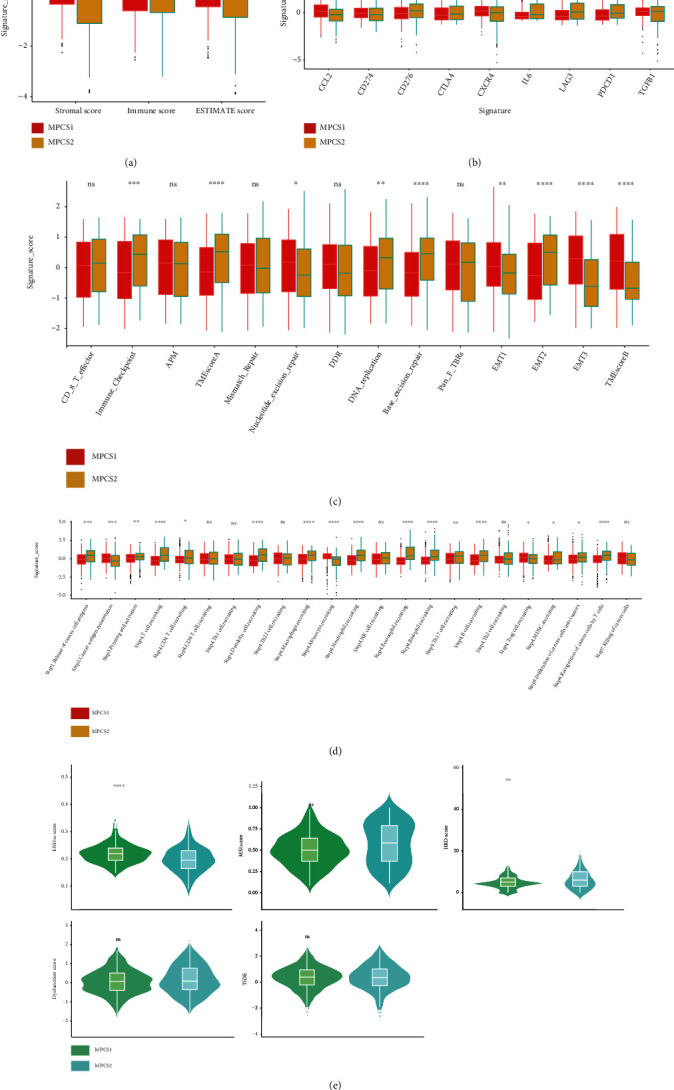
Landscapes of specific immune components and immune function scores for both subtypes. (a) ESTIMATE scores between MPCS1 and MPCS2. (b–d) The immune checkpoint inhibitors, normalized enrichment scores of immune effectors, and anticancer immune steps between MPCS1 and MPCS2. (e) EHN, MSI, HRD, dysfunction, and TIDE score between MPCS1 and MPCS2.

Immunotherapy combining targeted therapy has become first-line treatment for ccRCC in recent years. To delineate the immune infiltration profiles, GSVA was used to compare immune activity between the subgroups. Overall, the MPCS2 subtype indicated activated immune factors compared with the MPCS1 subtype. The MPCS2 subtype expressed higher level of CCL5, XCL1, CCL20, CXCL1, CXCL13, TGFBR1, IL6, CD28, CD80, CD27, and TNFSF9 (Figure 4(a)). Similarly, TME analysis found the MPCS2 contained higher levels of immune cell infiltration signatures, such as T cell regulatory (Tregs) (Figure 4(b)). Correspondingly, using the ESTIMATE algorithm, we observed that the MPCS2 subtype gained a higher stromal score and lower immune score than MPCS1 (Figure 5(a)). Notably, elevated immune infiltration often predicts poor prognosis in ccRCC patients owing to immune disorders. Hence, we analyzed specific immune components and immune steps in the anticancer process. The MPCS2 subtype was impaired in CD274, CCL2, Th cell, and DNA repair ability (Figures 5(b) and 5(c)). Of interest, the MPCS2 subtype reserved higher CD8 T effector levels, indicating its immunotherapeutic potential. Although MPCS2 contained higher immune infiltration, MPCS2 cannot effectively exert antitumor effect owing to lack of efficient immune cell recruitment (Figure 5(d)). Additionally, the MPCS1 subtype had a higher ENHss score, while the MPCS2 subtype acquired a higher RNAss score, microsatellite instability (MSI) score, and HRD score. These results indicated a stronger stemness ability and higher genomic instability of the MPCS2 subtype (Figure 5(e)). Regarding the dysfunction score and tumor immune dysfunction and exclusion (TIDE) score, no significant differences were observed between the two groups. The above findings suggested that melatonergic system axis signaling may be involved in the formation of distinct immune expression profiles between subgroups.

### 3.5. Comprehensive and Integrated Genomic Characteristics between the Two Subtypes

**Figure 6:**
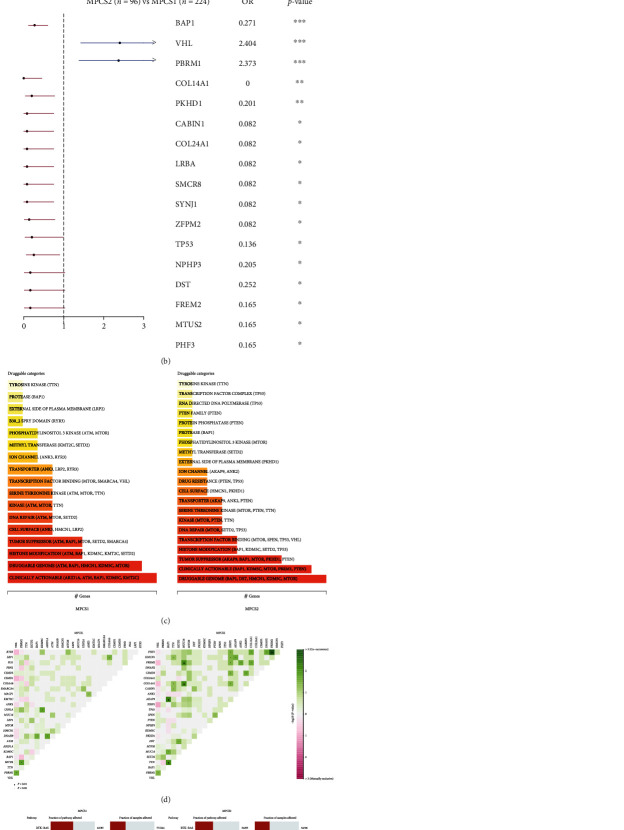
Profiles of somatic mutations between the two subtypes. (a) Mutational landscape. (b) Forest plot showing the prognostic impact of mutated signatures. (c) Potential druggable categories. (d) Comutation and coexisting mutation pattern. (e) Oncogenic signaling pathways in MPCS1 and MPCS2.

We also explored the divergence in genomic mutation between the subgroups except immune infiltration profiles. We found that MPCS1 showed a slightly higher mutation frequency than MPCS2 (87.05% vs. 86.46%). The waterfall charts listed the top 20 signatures owning the highest mutation frequency in MPCS1 and MPCS2 (Figure 6(a)). Of interest, BAP1, TTN, SETD2, MUC16, MTOR, and DST were more frequently mutated in MPCS2 than in MPCS1. We found that most of the mutated genes were detrimental to MPCS2 (Figure 6(b)). Based on the mutation data, potential targets of subtypes were probed using the DGIdb database. The druggable genes of the two subtypes were categorized into 17 categories and 21 categories. Most of the druggable genes were enriched in druggable genome and clinically actionable (Figure 6(c)). Somatic interaction analysis revealed a comutated model of KDM5C-USH2A and PBRM1-SETD2 in MPCS1 and BAP1-AKAP9, BAP1-TTN, and FREM2-PHF3 in MPCS2 (Figure 6(d)). Comutations suggested that they may act synergistically in the same pathway, with the selective advantage of retaining more mutations between them. In addition, R package Maftools was used to find enriched mutations in oncogenic pathways, including RTK-RAS, NOTCH, and PI3K. TP53 and PI3K were significantly affected in MPCS1, while PI3K and RTK–RAS were easily disturbed in MPCS2 (Figure 6(e)). Regarding mutations of melatonin regulators, compared to MPCS1, MPCS2 subtypes are more frequently mutated, of which CLOCK was the gene with the highest mutation frequency (Figure S4A).

**Figure 7:**
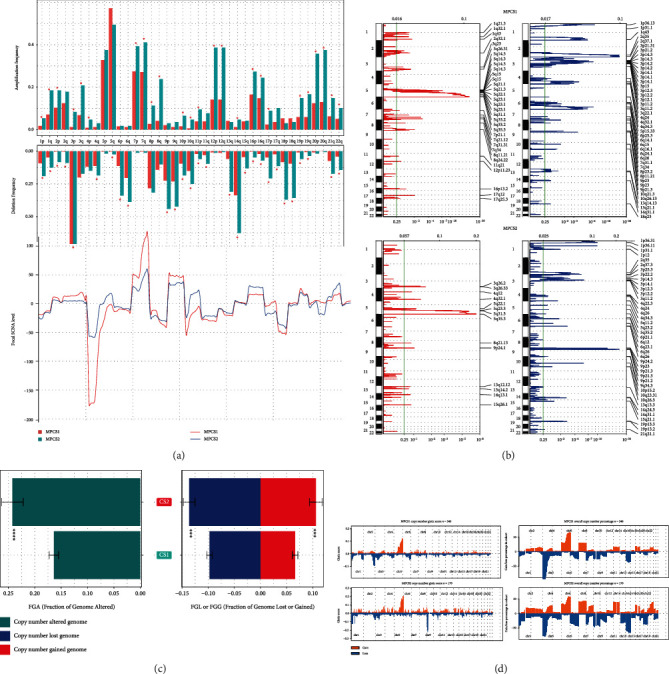
CNV of the two subtypes. (a) Comparison of amplification or deletion event between subtypes. (b) Specific amplification or deletion sites. (c) Bar plot indicating the total alteration frequency. (d) Detailed genomic gain or loss frequencies of CNV.

Copy number variations were also integrated between the two subtypes. Overall, the MPCS2 subtype conserved higher CNV frequencies both in the copy number loss genome and copy number gain genome than MPCS1 subtype (Figure 7(a)). We exhaustively decoded CNV amplifications and deletions on chromosomes using GISTIC2.0 software (Figures 7(b)–7(d), Table S3). As expected, the copy number GISTIC score and copy number percentage presented similar patterns in MPCS1 and MPCS2. Notably, both the MPCS1 and MPCS2 subgroups included frequent CNVs in Chr3, Chr5, and Chr7. The recurring CNVs of MPCS1 were amplification of 5q35.3 (CANX, CLTB) and 5q33.2 (KIF4B) and deletion of 2q37.1 (ALPI, CHRND) and 2q35 (MREG). The repeated CNVs of MPCS2 indicated the amplification of 5q35.3 (BNIP1, CANX) and 4q32.1 (ETFDH, RAPGEF2) and censoring of 9p23 (PTPRD) and 9p21.3 (CDKN2A) (Table S3). The distinctive CNV events may shape different melatonin-related molecular subtypes.

### 3.6. Drug Sensitivity Analysis of the Two Subtypes

**Figure 8:**
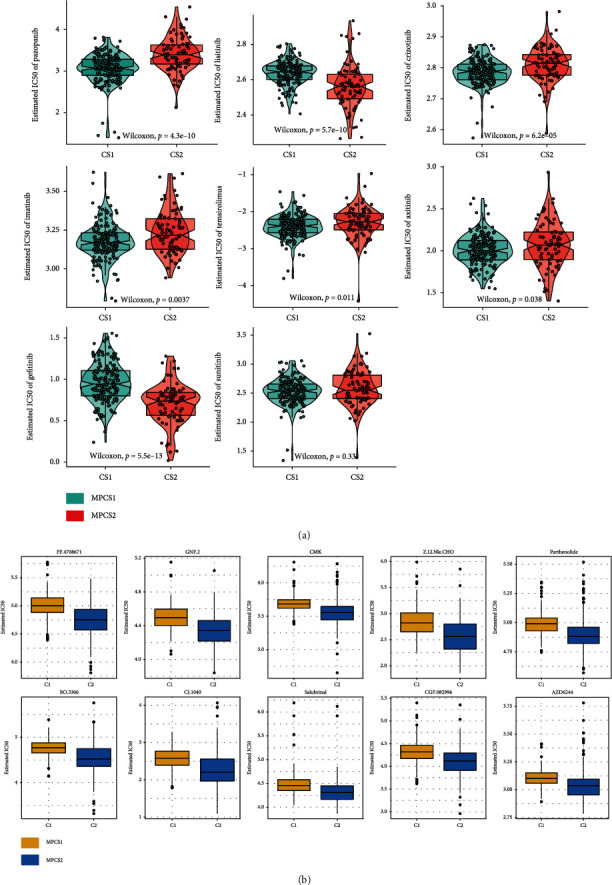
Drug sensitivity comparison of the two subtypes. (a) Distribution of IC50 values of chemotherapy agents. (b) Novel identified molecular agents for MPCS2 from the GDSC database.

The GDSC database was used to inspect potential drugs for MPCS1 and MPCS2. As expected, most of targeted drugs were more effective against MPCS1 subtype, the melatonin-active tumor subtype (Figure 8(a)). MPCS1 presented good response to pazopanib, linsitinib, crizotinib, imatinib, temsirolimus, and axitinib, while the MPCS2 subtype was more sensitive to gefitinib (Figure 8(a)). In addition, we further tested the drug reactivity of the two subtypes to 138 small molecule inhibitors (Table S4). Considering MPCS2 as a drug-refractory molecular subtype, we showed the top ten notable drugs for the MPCS2 subtype (Figure 8(b)). The MPCS2 subtype exhibited sensitivity to PF.4708671, GNF.2, CMK, Z.LLNle.CHO, and Parthenolide. Conceivably, PF.4708671 and GNF.2, the direct inhibitors of S6K1 and RSK2 pathway, could provide effective targets against MPCS2 subtype.

### 3.7. Reliability of MPCS1 and MPCS2 in the External Dataset

**Figure 9:**
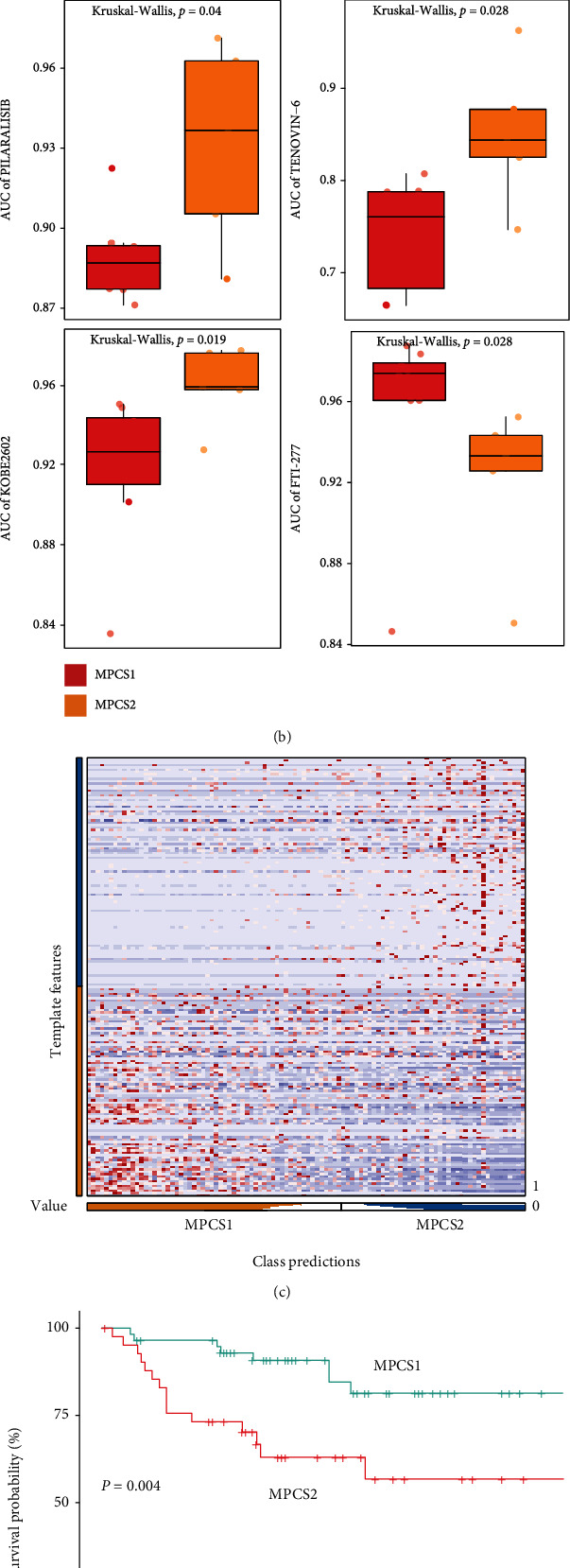
Verification of the remodeling system via external cohorts. (a) Heatmap illustrating the expression pattern of melatonin regulators in ccRCC cell lines. (b) Drug susceptibility assessments were performed using standardized AUC. (c) Heatmap of biomarker expression pattern. (d) Survival analysis of the two predicted subtypes in JAPAN-KIRC.

We used the GDSC database and JAPAN-KIRC cohort to approve the reliability of the subtyping model. Significant differences were observed between the two subgroups after classification of ccRCC cell lines (Figure 9(a)). Consistent with TCGA analysis results, we found that most melatonin regulators were downregulated in the MPCS2 group. The AUC was calculated to evaluate drug sensitivity between subgroups. Not surprisingly, the MPCS2 subgroup achieved higher AUCs in almost all drugs (Figure 9(b)). MPCS1 subgroup presented sensitivity to pilaralisib, tenovin−6, kobe2602, METAP2 inhibitor, IKK–2–inhibitor–V, palbociclib, dacinostat, AT7867, and filanesib, while the MPCS2 subtype was only sensitive to FTI−277 (Figure 9(b) and Figure S4B). Using NTP algorithm, the biomarkers (higher expressed genes in each subtype) from TCGA-ccRCC could divide the JAPAN-KIRC cohort into two different subgroups (Figure 9(c)). Consequently, MPCS2 subgroup patients indicated poorer survival than MPCS1 subgroup, in keeping with TCGA-KIRC data (Figure 9(d)). The above finding recognized the reliability of the classification model.

### 3.8. Construction and Validation of the Novel Risk Model of Melatonin

**Figure 10:**
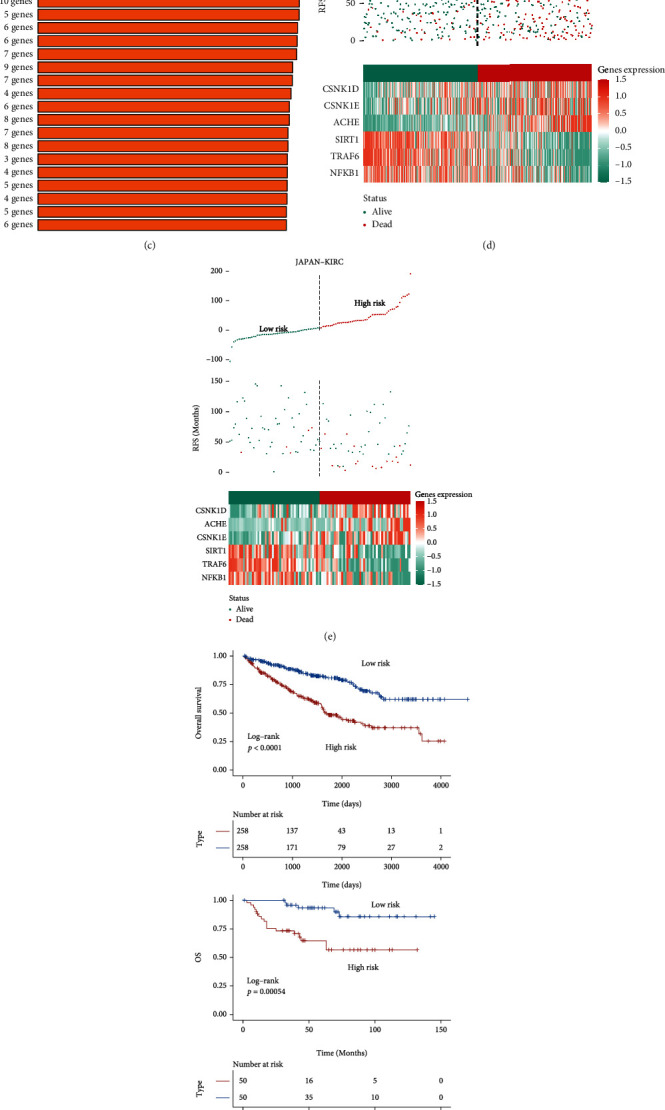
Construction and verification of a novel risk model. (a) Volcano plot illustrating the prognostic impact of biomarkers. (b) Random forest ranking the importance of the top 10 signatures. (c) The number of signatures' combination and each model's *p* value. (d, e) Risk score distribution plot divided patients from TCGA-ccRCC and JAPAN-KIRC cohorts into high- and low-risk groups. (f) Survival analysis of the two risk groups in TCGA-ccRCC and JAPAN-KIRC cohorts. (g, h) The time-dependent ROC curves for the two risk groups in TCGA-ccRCC and JAPAN-KIRC cohorts.

Since melatonin-associated subtypes displayed distinct clinical and molecular features, we then constructed a prognostic risk model based on subtype biomarkers. We identified significantly altered genes between subtypes using univariate Cox regression analysis (Figure 10(a)). We next screened the ten most relevant genes and performed random gene combinations and Kaplan-Meier (KM) analysis to measure *p* values for each model (Figures 10(b) and 10(c)). As a result, the optimal risk model composed of six genes (CSNK1D, CSNK1E, ACHE, SIRT1, TRAF6, and NFKB1) was screened out and named RCC-MP6 (Figure 10(c)). Each RCC patient's risk score was calculated by formula as follows: RCC‐MP6 = 3.486773^∗^ CSNK1D + 3.893991^∗^ CSNK1E + 5.439769^∗^ ACHE − 4.196123^∗^ SIRT1 − 5.293665^∗^TRAF6 − 3.462471^∗^ NFKB1. Based on the median value, the RCC-MP6 model could effectively classify TCGA-ccRCC and JAPAN-KIRC into high-risk and low-risk groups, respectively (Figures 10(d) and 10(e)). We found that the high-risk subgroup displayed inferior clinical outcome than the low-risk group (Figure 10(f)). The satisfactory AUC scores were obtained in TCGA-ccRCC cohort, and AUC scores were 0.697, 0.688, and 0.725 at 1 year, 3 years, and 5 years, respectively (Figure 10(g)). The RCC-MP6 model achieved better predictive value in the JAPAN-KIRC dataset, since all the AUC scores of 1-, 2-, 3-, and 5-year OS exceeded 0.7 (Figure 10(h)). By comparison with clinical factors, including age and stage, the RCC-MP6 risk score showed a higher sensitivity and specific predictive ability (Figure S5A). Furthermore, the calibration curves showed a satisfactory consistent fit between the predicted and observed values for 1-, 2-, 3-, and 5-year OS in both TCGA-KIRC and JAPAN-KIRC (Figure S5B).

### 3.9. Impact of ACHE on ccRCC Immunity

**Figure 11:**
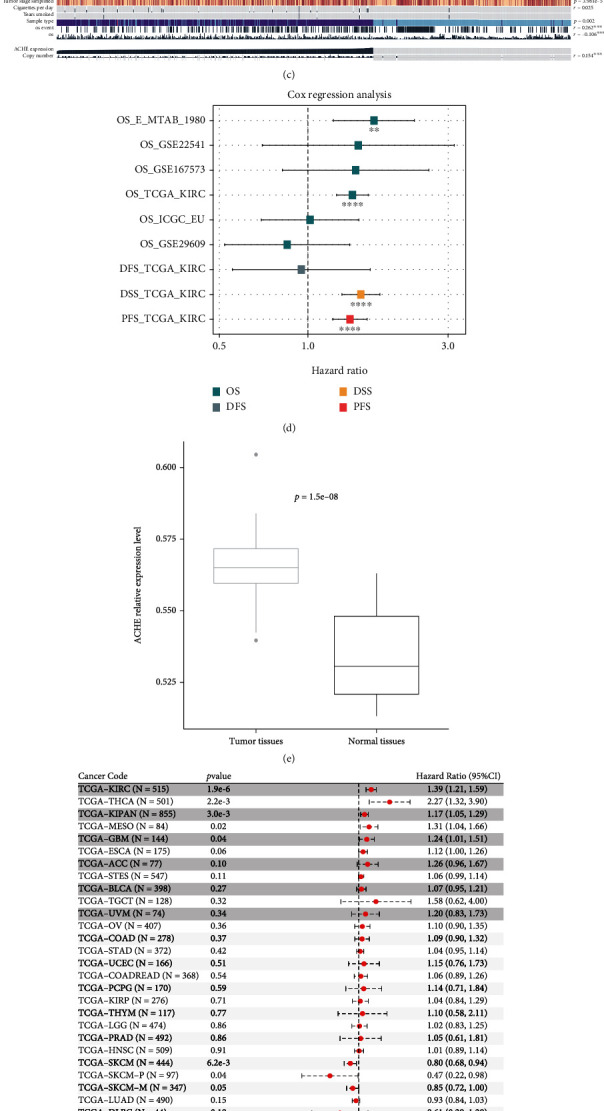
Impact of ACHE in ccRCC and pan-cancer. (a) Radom forest tree showing the importance of melatonin-related signatures. (b) Different expression levels of ACHE in ccRCC. (c) Correlation of the ACHE expression level and clinical characteristics in TCGA-KIRC. (d) Impact of ACHE on survival in ccRCC. (e) Verification of ACHE expression in the Changhai cohort. (f, g) Cox analysis and biological analysis of ACHE across cancers. (h) Impact of mutation state of ACHE on ccRCC immunity.

Considering the impact of melatonin regulators in ccRCC, we then aimed to investigate which regulator led to the highest proportion of prognostic importance. Utilizing the random forest algorithm, we observed that ACHE might display the key role in the melatonergic system in ccRCC (Figure 11(a)). Regarding expression level, ACHE was more highly expressed in tumor tissues and correlated with inferior clinical outcome in ccRCC (Figure 11(b)). The ACHE expression level was also related to clinical characteristics, including age, Karnofsky performance score, and copy number (Figure 11(c)). In addition to TCGA-KIRC cohort, high ACHE expression level also indicated poor prognosis in the E-MTAB-1980 cohort (Figure 11(d)). We verified the differential expression of ACHE in Changhai cohort (Figure 11(e)). Among pan-cancers, ACHE could be treated as specific prognostic biomarker for KIRC and THCA (Figure 11(f)). As for the biological function of ACHE in ccRCC, Figure 11(g) shows that a high level of ACHE could activate KRAS, interferon-*γ* and interferon-*α*, inflammatory IL6-JAK-STATS and IL2-STAT5 pathways in pan-cancer, especially in ccRCC, which indicated that ACHE was involved in immune regulation in cancer. ACHE mutation states were significantly related to infiltration degree of CD4^+^ T cells in ccRCC (Figure 11(h)).

## 4. Discussion

ccRCC patients with close pathological features may show completely different prognoses owing to the existence of cancer heterogeneity [35]. It is necessary to accurately predict patient survival based on the characteristics of subtypes. Melatonin axis signaling was broadly involved in cancer development and progression [36]. Due to its antioxidant, anti-inflammatory, and immunomodulatory activities, melatonin reduces the morbidity and associated mortality of fatal viral infections, including COVID-19 [37]. The relationship between melatonin and ccRCC was not yet understood. Some studies have focused on single-molecule regulatory mechanisms; the global signature induced by melatonergic system axis signaling has rarely been explored. Nonetheless, melatonin-based molecular subtypes in tumor repertoires have rarely been explored. In this study, we made a comprehensive summarization of melatonin regulator genes via in silico analysis. We found that those signatures were significantly dysregulated across pan-cancer and were associated with genomic and epigenetic modification. According to the expression pattern of those signatures, ccRCC patients could be subgrouped into two significantly different melatonin-associated subtypes (MPCS1 and MPCS2). Compared with MPCS1, the MPCS2 exhibited activated metabolic profile, immune disorder feature, higher tumor mutation burden, and poorer survival. Furthermore, we established a reliable risk model based on subtype biomarkers and validated the model using external datasets.

Melatonergic system axis signaling not only regulates circadian rhythms but also exerts anticancer activities. We observed that the DEGs between the two subgroups were enriched in the immune UV response, programmed cell death, and acute inflammatory response. Goradel et al. reported that melatonin functioned as a suppressor of breast cancer angiogenesis by downregulating the expression of HIF-1 [38]. Some cytokines such as IFN, GM-CSF, and G-CSF could stimulate the secretion of melatonin, while IL-1 did the opposite [39]. It has been found that melatonin treatment significantly affects macrophages and monocytes, promoting the release of proinflammatory cytokines. The MPCS2 subtype was found to be activated in metabolic pathways. The activated metabolic environment was able to support tumor expansion. Of interest, the melatonin-suppressed phenotype of MPCS2 suggested the possibility of utilizing melatonin to inhibit tumor progression. A recent study indicated that melatonin was able to regulate tumor angiogenesis through the miR-424/VEGFA signaling axis in osteosarcoma [40]. Melatonin prevented angiogenesis of HepG2 by inhibiting the HIF-1/STAT3 pathway [41]. In fact, melatonin counteracts oxidative stress-induced apoptosis and enhanced the effects of autophagy [42]. A protective effect of melatonin on the testes was attributed to its ability to activate SIRT1, which alleviated the effects of oxidative stress, mitochondrial dysfunction, and DNA damage [43]. Thus, targeting the melatonergic system axis may have a synergistic antitumor effect with targeted therapy or immunotherapy.

Recently, the combination of immunotherapy and targeted therapy has been used as a first-line treatment for metastatic ccRCC. However, frequent drug resistance limits the efficacy of immune drugs. Melatonin axis regulation is also involved in immune environment. The MPCS2 subtype expressed higher levels of immune components, such as CCl5 and IL6. Huang reported that TAMs produce CCL5, which activates the I-CATENIN/STAT3 pathway, potentially contributing to prostate cancer stem cell survival and metastasis [44]. Cho et al. found that cancer-associated fibroblasts (CAFs) enhanced monocyte differentiation and promoted TAM activation by IL6 and GM-CSF factors [45]. Treg cells are key factors in immune escape and were higher enriched in the MPCS2 subtype. We found that the MPCS2 subtype was impaired in CD274 (PD-L1), CCL2, Th cell, and DNA repair ability. PD-L1 is a key target of immune escape, and its dysregulation may be involved in inducing immune dysfunction in the MPCS2 subtype. Leem et al. proposed that melatonin protected mouse oocytes from DNA damage by strengthening nonhomologous end joining repair [46]. Actually, the rather unique capability of melatonin to display multiple neutralizing actions against diverse threatening roles, melatonin is significant to its role in preventing oxidative damage to DNA [47]. We found that the MPCS2 subtype retained higher CD8 T effector levels, indicating its immunotherapeutic potential. Thus, activation of the melatonergic system axis may enhance the efficacy of immunotherapy in the treatment of ccRCC.

The response to immunotherapy was based on genome mutations. Melatonin regulator genes were involved in genome mutations. BAP1, TTN, SETD2, MUC16, MTOR, and DST were frequently mutated in MPCS2. Loss of BAP1 leads to growth inhibition of renal cancer cells and enhances the effect of mesenchymal–epithelial transition [48]. Wang et al. reported that TTN and MUC16 exhibited higher mutation frequencies in a high immunity group of colon cancer [49]. Kanu et al. found that inactivation of SETD2 promoted kidney cancer branch evolution through replication stress and impaired DNA repair [50]. Everolimus is an MTOR-targeted drug that plays an important role in the targeted therapy of renal cancer. PI3K and RTK–RAS were easily disturbed in the MPCS2 subtype. Tan and his colleagues found that N6-methyladenosine modification of lncRNA DUXAP9 promoted renal cancer cell proliferation migration and invasion by boosting the PI3K/AKT signaling [51]. For copy number variations, the MPCS2 subtype conserved higher CNV frequencies than the MPCS1 subtype. Recent reports also found that most of the samples tested had CNV deletion of chromosome 3p (95%) and CNV gain of 5q (72%) [52]. They also detected a high frequency loss of 9p and 14q (65%, 68%), which are regarded as hallmarks of ccRCC metastasis [53]. Moreover, Zhou et al. found that the circadian clock may play a role in regulating the microenvironment in ccRCC tumors [54]. Therefore, melatonin modulators may interact with genomic mutations to induce tumor heterogeneity.

Different melatonin-associated subtypes showed significantly different drug responses. The MPCS2 subtype, the drug-refractory group, was only sensitive to gefitinib. We further explored the potential molecular inhibitors for subtype. PF.4708671 and GNF.2, direct inhibitors of the S6K1 and RSK2 pathways, indicated good performance for MPCS2. Notably, a reliable risk model RCC-MP6, based on CSNK1D, CSNK1E, ACHE, SIRT1, TRAF6, and NFKB1, was constructed to predict patient prognosis. Casein kinase 1 members are involved in a variety of cellular growth and survival processes, including circadian rhythms and DNA repair. Besides its role in synaptic transmission, ACHE also regulates multiple oncogenic signaling pathways involved in the classic function of tumors, including proliferation, invasion, and metastasis [55]. A study by Tan et al. showed that SRT1720 inhibits bladder cancer growth by inhibiting the SIRT1-HIF pathway [56]. Meng et al. found that blocking miR-146b-5p inhibited tumor growth and enhanced inflammatory response by increasing TRAF6 expression [57]. The effectiveness of RCC-MP6 was also confirmed using external datasets. Nonetheless, more datasets are needed in the future to verify the effectiveness of the model.

In this work, we systematically explored the function of melatonin regulator genes and identified two molecular subtypes of ccRCC. There are also some limitations of our study. First, the main findings were based on bioinformatics analyses, which need further verification in other datasets. Second, the risk model may be disturbed by some confounding factors, such as area, race, gender, comorbidities, smoking, comorbidities, and family history. Thus, independent and prospective datasets are warranted to confirm this risk model.

In summary, activating melatonergic system axis signaling may be an appropriate approach for ccRCC treatment. The melatonergic system axis may alter the immunosuppressive microenvironment and promote antitumor immune responses. Hopefully, our study will improve our understanding of melatonin's relationship with ccRCC and provide clinical reference to predict prognosis.

## Data Availability

The datasets presented in the study are included in Materials and Method. Further inquiries can be directed to the corresponding authors.

## Supplementary Materials

Supplementary Materials Figure S1: flowchart of this study. Figure S2: genome mutation spectrum of melatonin regulators across cancers. (A) CNV events of melatonin-related signatures in the TCGA-ccRCC dataset. Blue represents CNV loss; red represents CNV gain; green represents no CNV. (B) Mutation frequency and type of melatonin regulators in multiple cancers. (C) Comparison of mutation frequencies in melatonin-regulated genes in multiple cancers. Figure S3: complementary enrichment analysis. (A) Circle plot indicating the BP and MF GO enrichment analysis of DEGs. (B) Heatmap illustrating the different activated score of metabolism-related signatures. Figure S4: validating the classification model with external datasets. (A) Waterfall plot showing the mutation paradigm of melatonin regulator genes in MPCS1 and MPCS2. (B) Drug susceptibility assessments were performed based on standardized AUC using the GDSC database. Figure S5: sensitivity and specify of RCC-MP6. (A) The decision curve analysis for RCC-MP6 evaluating ccRCC patient with 1-, 2-, 3-, and 5-year overall survival in TCGA-KIRC (left) and JAPAN-KIRC (right) cohorts. (B) The calibration plot of RCC-MP6 for examining the probability of 1-, 2-, 3-, and 5-year overall survival in TCGA-KIRC (left) and JAPAN-KIRC (right) cohorts. Table S1: list of melatonin regulators. Table S2: differences in the clinical characteristics of different subtypes. Table S3: recurrent amplification and deletion regions between subgroups. Table S4: list of small molecule drugs validated for analysis. Table S5: abbreviations.
