# Supplementary material for: Deciphering the Role of Melatonin-Related Signatures in Tumor Immunity and the Prognosis of Clear Cell Renal Cell Carcinoma
Source: Oxid Med Cell Longev. 2023 Feb 14;2023:3077091. doi: 10.1155/2023/3077091 (PMC9943605; doi:10.1155/2023/3077091)
Supplement: Supplementary Materials — Figure S1: flowchart of this study. Figure S2: genome mutation spectrum of melatonin regulators across cancers. (A) CNV events of melatonin-related signatures in the TCGA-ccRCC dataset. Blue represents CNV loss; red represents CNV gain; green represents no CNV. (B) Mutation frequency and type of melatonin regulators in multiple cancers. (C) Comparison of mutation frequencies in melatonin-regulated genes in multiple cancers. Figure S3: complementary enrichment analysis. (A) Circle plot indicating the BP and MF GO enrichment analysis of DEGs. (B) Heatmap illustrating the different activated score of metabolism-related signatures. Figure S4: validating the classification model with external datasets. (A) Waterfall plot showing the mutation paradigm of melatonin regulator genes in MPCS1 and MPCS2. (B) Drug susceptibility assessments were performed based on standardized AUC using the GDSC database. Figure S5: sensitivity and specify of RCC-MP6. (A) The decision curve analysis for RCC-MP6 evaluating ccRCC patient with 1-, 2-, 3-, and 5-year overall survival in TCGA-KIRC (left) and JAPAN-KIRC (right) cohorts. (B) The calibration plot of RCC-MP6 for examining the probability of 1-, 2-, 3-, and 5-year overall survival in TCGA-KIRC (left) and JAPAN-KIRC (right) cohorts. Table S1: list of melatonin regulators. Table S2: differences in the clinical characteristics of different subtypes. Table S3: recurrent amplification and deletion regions between subgroups. Table S4: list of small molecule drugs validated for analysis. Table S5: abbreviations. [file 3077091.f1.zip › TableS5.docx]

**Abbreviation list**

ccRCC: clear cell renal cell carcinoma

MPCS: melatonin-pattern cancer subtype

TNM: tumor, node, and metastasis

ICI: immune check point inhibitor

RCC: Renal cell carcinoma

OXPHOS: oxidative phosphorylation

ROS: Reactive oxygen species

HIF: Hypoxia-inducible factor

DEG: differentially expressed gene

GO: Gene Ontology

KEGG: Kyoto Encyclopedia of Genes and Genomes

GSEA: Gene Set Enrichment Analysis

GEVA: Gene Set Variation Analysis

ssGSVA: single-sample gene set enrichment analysis

TIDE: Tumor Immune Dysfunction and Exclusion

RSFVH: Random Survival Forest Variable Hunting

RT-qPCR: quantitative reverse transcriptase-polymerase chain reaction

ACHE: Acetylcholinesterase

CNV: copy number variation

SNV: single-nucleotide variation

ACC: Adrenocortical carcinoma

BLCA: Bladder urothelial carcinoma

BRCA: Breast invasive carcinoma

CESC: Cervical squamous cell carcinoma and endocervical adenocarcinoma

CHOL: Cholangiocarcinoma

COAD: Colon adenocarcinoma

DLBC: Lymphoid neoplasm diffuse large B-cell lymphoma

ESCA: Esophageal carcinoma

GBM: Glioblastoma multiforme

HNSC: Head and neck squamous cell carcinoma

KICH: Kidney chromophobe

KIRC: Kidney renal clear cell carcinoma

KIRP: Kidney renal papillary cell carcinoma

LAML: Acute myeloid leukemia

LGG: Brain lower-grade glioma

LIHC: Liver hepatocellular carcinoma

LUAD: Lung adenocarcinoma

LUSC: Lung squamous cell carcinoma

MESO: Mesothelioma

OV: Ovarian serous cystadenocarcinoma

PAAD: Pancreatic adenocarcinoma

PCPG: Pheochromocytoma and paraganglioma

PRAD: Prostate adenocarcinoma

READ: Rectum adenocarcinoma

SARC: Sarcoma

SKCM: Skin cutaneous melanoma

STAD: Stomach adenocarcinoma

STES: Stomach and esophageal carcinoma

TGCT: Testicular germ cell tumor

THCA: Thyroid carcinoma

THYM: Thymoma

UCEC: Uterine corpus endometrial carcinoma

UCS: Uterine carcinosarcoma

UVM: Uveal melanoma

PAC: proportion of ambiguous clustering

OS: overall survival

PFS: progression free survival

Tregs: T cell regulatory cells

AUC: area under the curve

ENHss: Enhancer Elements/ DNA methylation-based stemness score

MSI: Microsatellite instability

HRD: Homologous recombination deficiency

KM: Kaplan-Meier

IFN: Interferon

GM-CSF: Granulocyte-macrophage colony-stimulating factor

G-CSF: Granulocyte Colony Stimulating Factor

TAM: Tumor-associated macrophages
