# Supplementary figures and images for: Deciphering the Role of Melatonin-Related Signatures in Tumor Immunity and the Prognosis of Clear Cell Renal Cell Carcinoma
Source: Oxid Med Cell Longev. 2023 Feb 14;2023:3077091. doi: 10.1155/2023/3077091 (PMC9943605; doi:10.1155/2023/3077091)

Figure S1

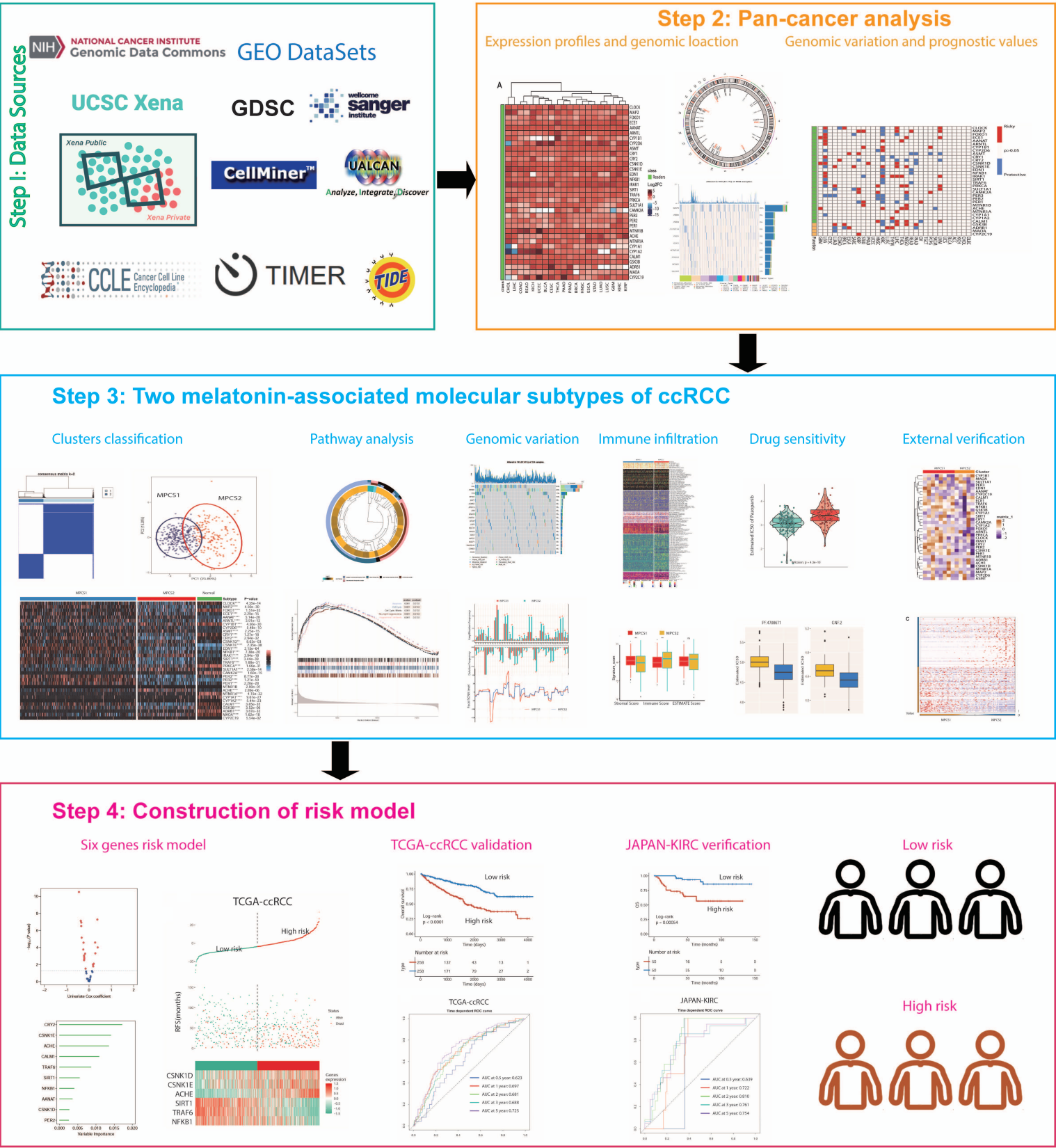

Figure S2

A

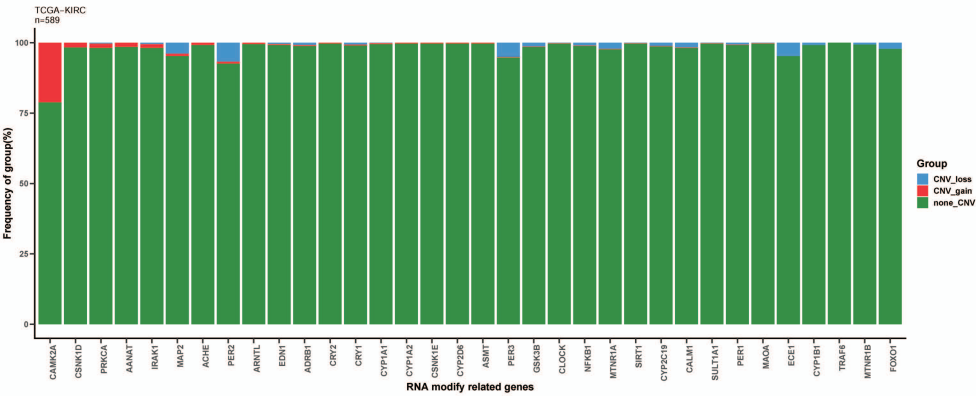

B

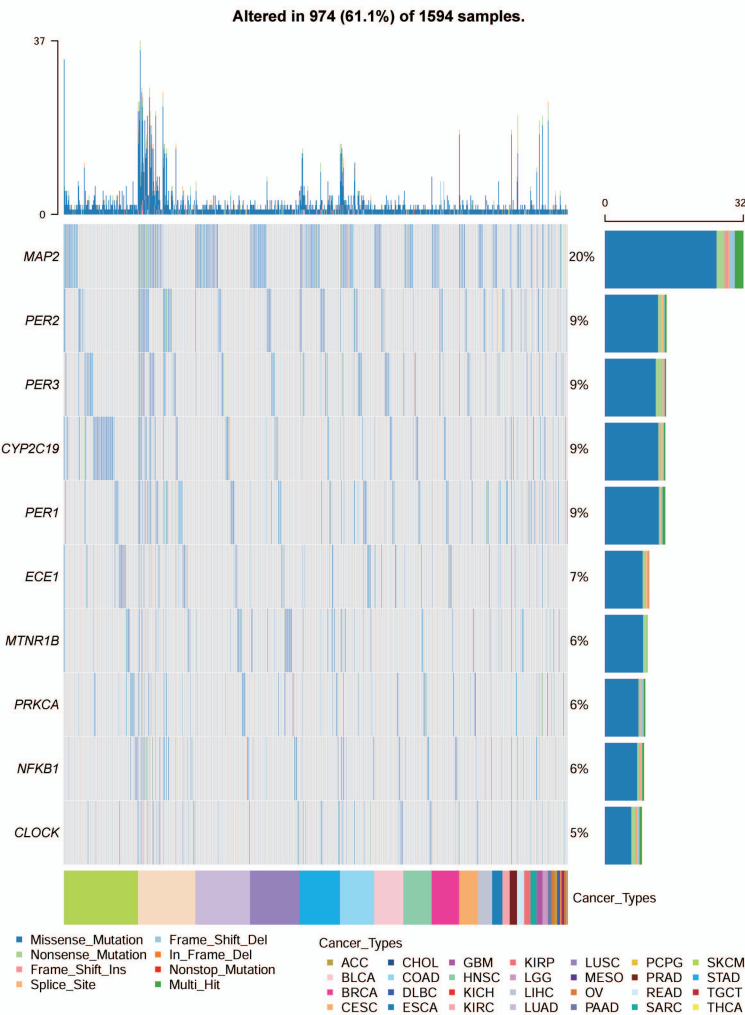

C

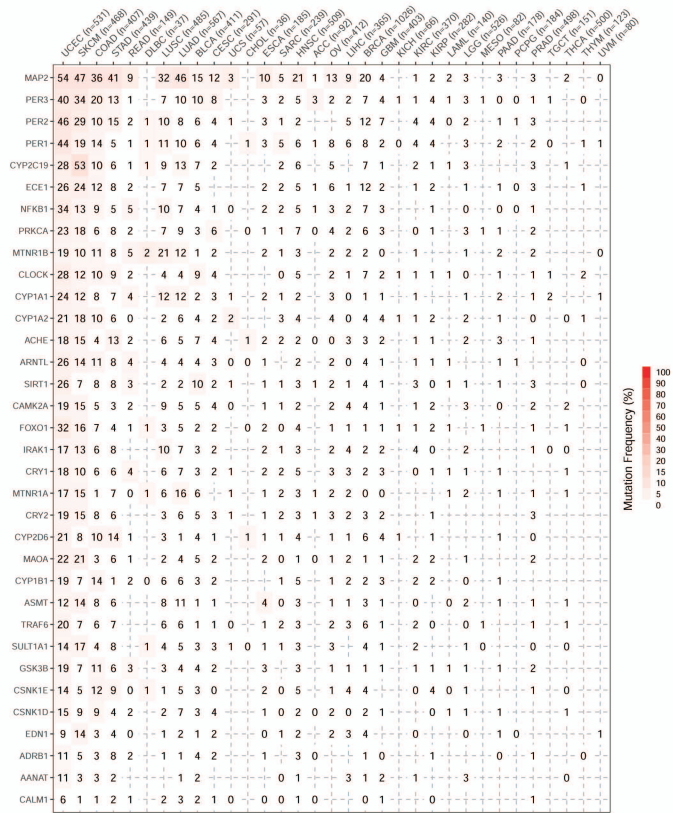

Figure S3

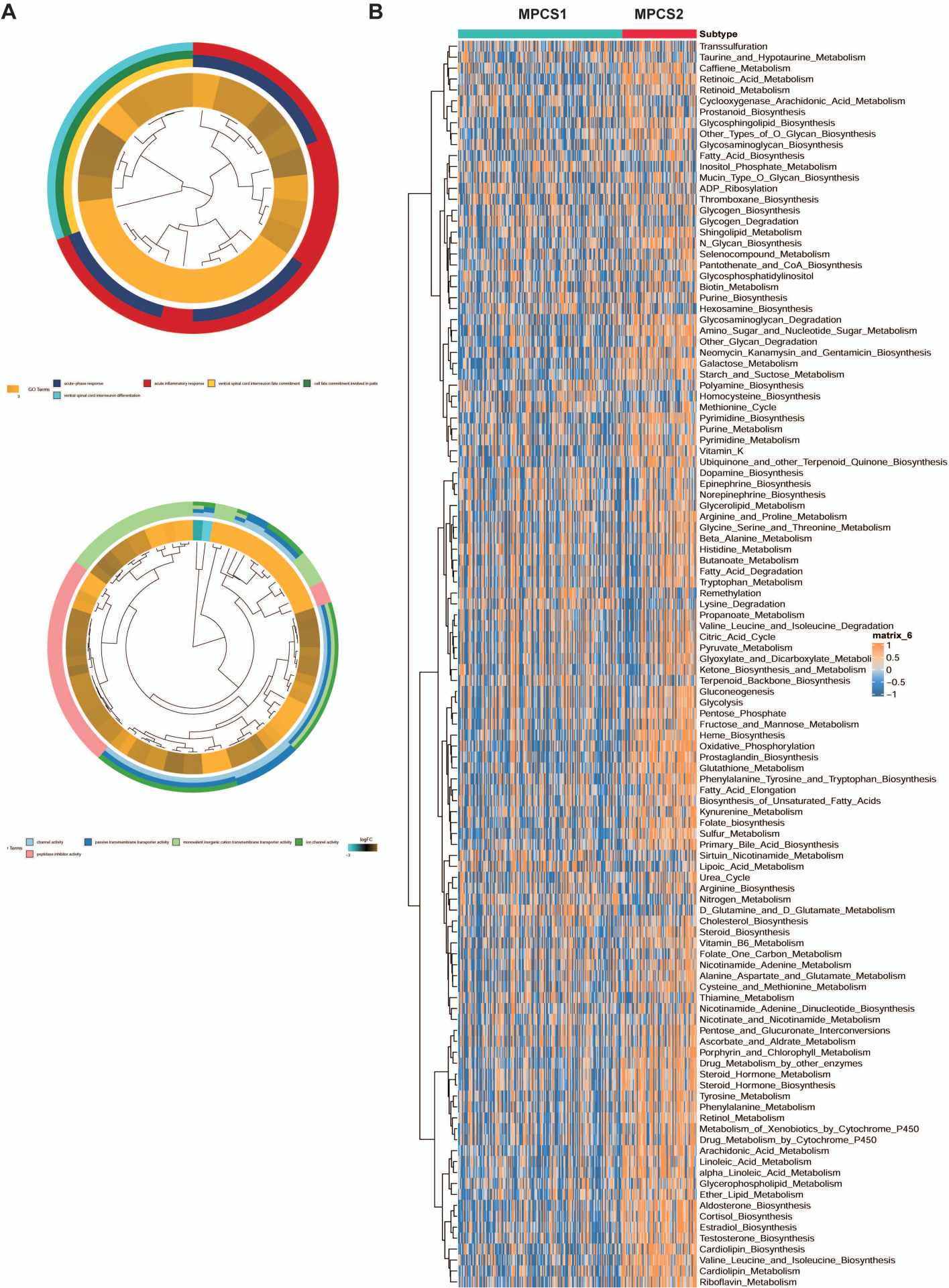

Figure S4

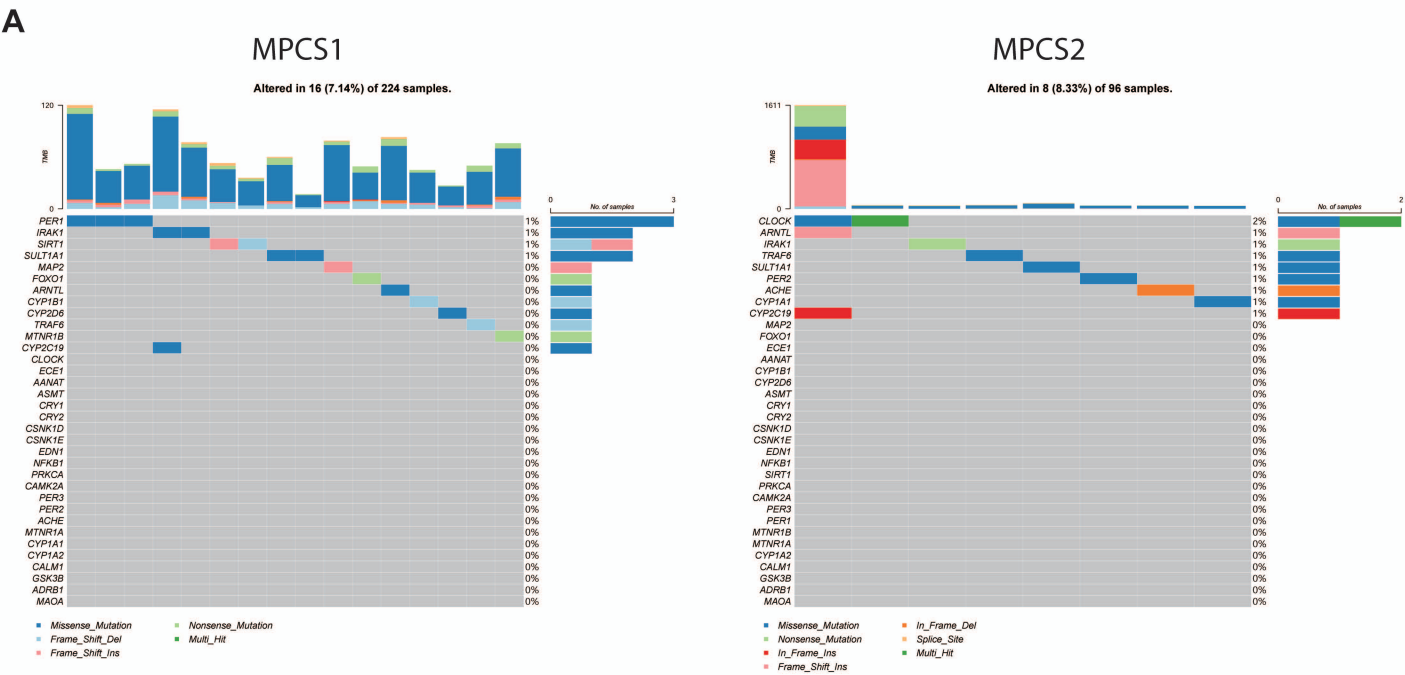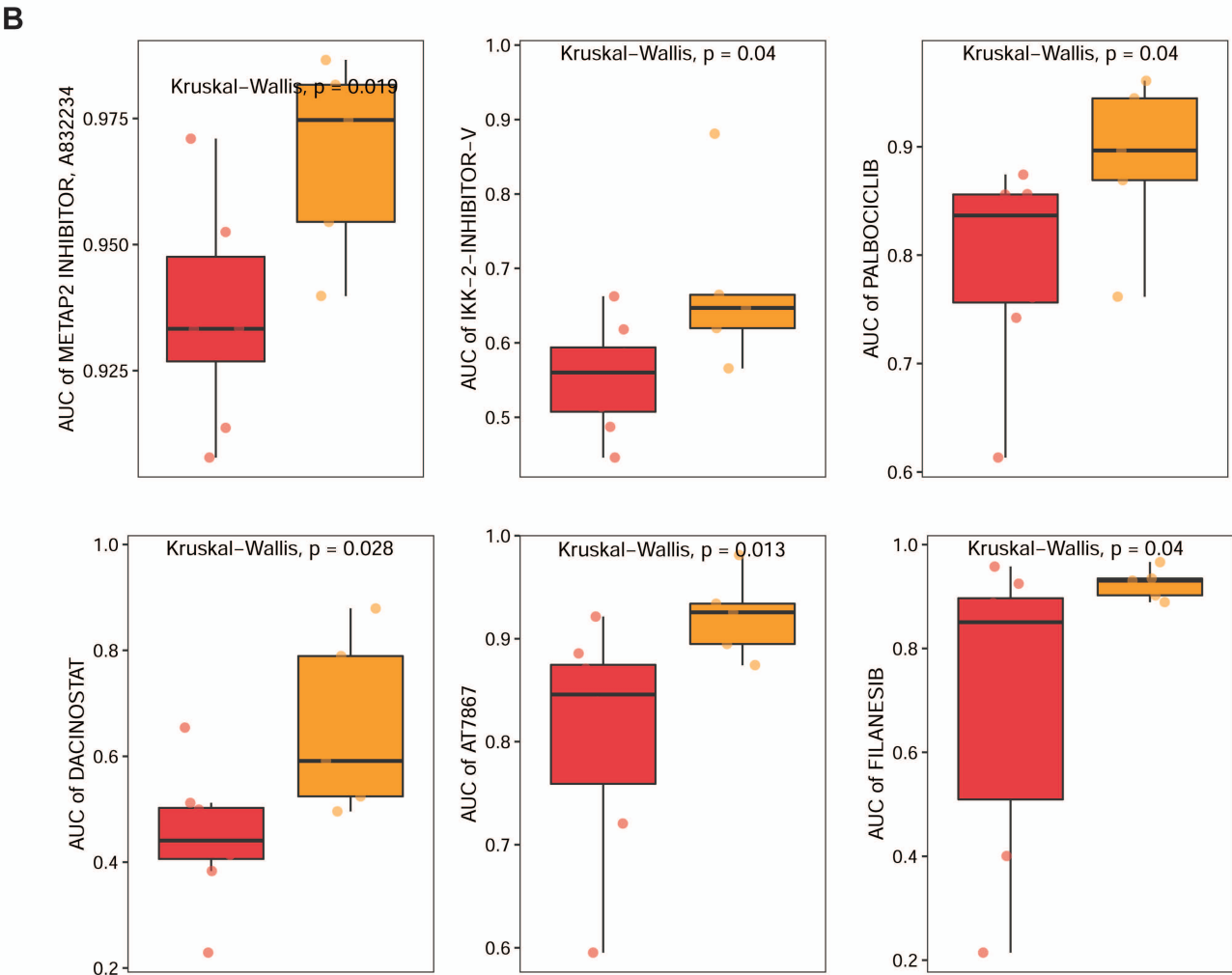

Figure S5

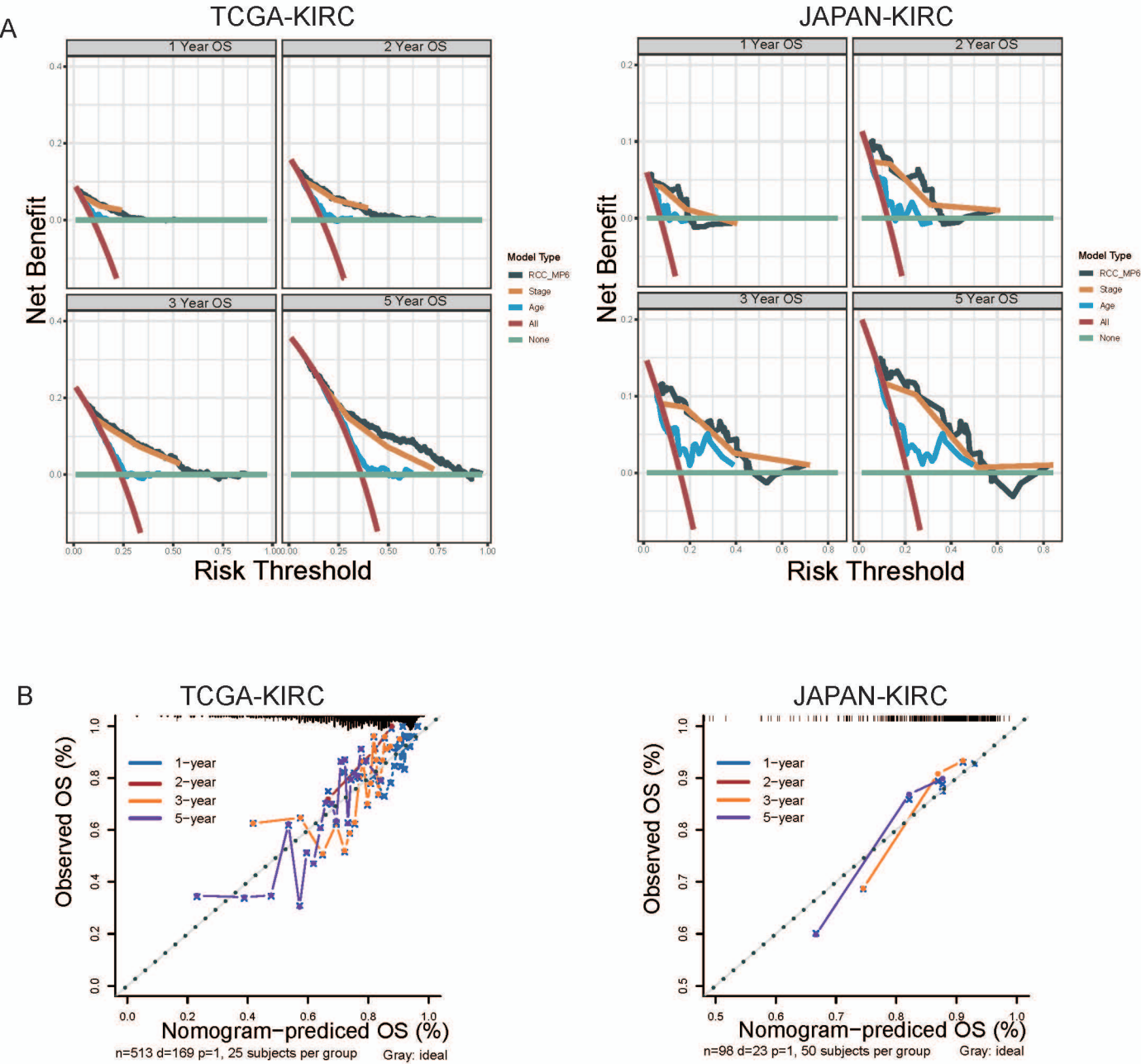

Supplement: Supplementary Materials — Figure S1: flowchart of this study. Figure S2: genome mutation spectrum of melatonin regulators across cancers. (A) CNV events of melatonin-related signatures in the TCGA-ccRCC dataset. Blue represents CNV loss; red represents CNV gain; green represents no CNV. (B) Mutation frequency and type of melatonin regulators in multiple cancers. (C) Comparison of mutation frequencies in melatonin-regulated genes in multiple cancers. Figure S3: complementary enrichment analysis. (A) Circle plot indicating the BP and MF GO enrichment analysis of DEGs. (B) Heatmap illustrating the different activated score of metabolism-related signatures. Figure S4: validating the classification model with external datasets. (A) Waterfall plot showing the mutation paradigm of melatonin regulator genes in MPCS1 and MPCS2. (B) Drug susceptibility assessments were performed based on standardized AUC using the GDSC database. Figure S5: sensitivity and specify of RCC-MP6. (A) The decision curve analysis for RCC-MP6 evaluating ccRCC patient with 1-, 2-, 3-, and 5-year overall survival in TCGA-KIRC (left) and JAPAN-KIRC (right) cohorts. (B) The calibration plot of RCC-MP6 for examining the probability of 1-, 2-, 3-, and 5-year overall survival in TCGA-KIRC (left) and JAPAN-KIRC (right) cohorts. Table S1: list of melatonin regulators. Table S2: differences in the clinical characteristics of different subtypes. Table S3: recurrent amplification and deletion regions between subgroups. Table S4: list of small molecule drugs validated for analysis. Table S5: abbreviations. [file 3077091.f1.zip › FigureS1-5.pdf]
